# Meeting of the American Medical Association

**Published:** 1863-04

**Authors:** 


### EDITORIAL DEPARTMENT.

### MEETING OF THE AMERICAN MEDICAL ASSOCIATION.

In our last number we had space only for the notice of the meeting o the *American Medical Association* to be held in June next. This notice was sent as by the Chairman of the Committee of Arrangements, Dr. N. S. Davis of Chicago. As is well known, this meeting of what has been properly called the National Medical Congress, has been postponed on account of the unhappy political divisions and excitements which were supposed to have separated those who were formerly attached by the interests of a common brotherhood.

If there is important business to be transacted, and the meeting of this Association will in any degree advance the interests of the profession, then we are glad that this call has been made, since we can see no good ground for supposing that delay would in any way restore or promote harmony in the profession in the different sections of the country. For the present, at least, our Southern brothers have withdrawn from us their sympathies, and come to regard with fixed opposition every measure which can promote our real prosperity; in the excitements of political and sectional contest they do not recognize the higher and holier bonds which have, and ever should, unite the members of the medical profession.

If it was possible for them to do so, it is probable that the members of the Association from the South, will not for the present care to participate in the proceedings of the Association; they propose "independence in government, religion, science and medicine," and with their politicians and people generally have grown mad, and desire nothing in common with us.—This, however, constitutes no reason for postponing the meeting of the Association, or any ground for discouragement in the members from the loyal States. Returning peace, with its seasons of calm reflection will not, we trust, fail to restore again the professional fellowship we have so long enjoyed.

So far as we are informed, it does not yet fully appear what is to be the exact order of exercises or business at this proposed meeting of the Association; various topics of interest have however been suggested, prominent among them the consideration of the establishment of a school of Military Surgery, elevation of the rank of the Medical Staff of the Army, and uniform standard of medical education. It is not proper to discuss these subjects too freely until it appears by what plan of procedure it is proposed to organize such school or obtain uniform and improved standard of medical education, though these questions are of great interest to the profession and to the country. Other governments may have their schools of Medical Surgery in successful and efficient operation, and with them they may be necessary and proper; but with us, where private enterprise is so much more active and powerful than governmental forces, the necessity for such institutions cannot be said to exist, and if we should ever see instituted in this country a Government School for the education of Military Surgeons, we shall also see another political organization, conducted by political men, nourished by political favor, and distinguished upon all occasions by want of everything except political merit. If in the present condition of the country, with our necessity for military surgeons and the great numbers of young physicians who are seeking the army for employment, competent, thorough instruction is not given in all our established medical schools in the minute details of military practice, then they are no longer worthy the confidence and support of the profession, and it will require no prophetic gift to foretell the doom of any institution where this branch is neglected. This is a necessity which will "create a supply," and no anxiety need be entertained upon the subject; while a higher standard of education, more rigid tests of excellence and competency are subjects which will properly engage the attention of all who are interested in the good of the profession or welfare of the country.

Since writing the above, mainly for the purpose of drawing attention to the meeting of the Association, we have received the March number of the *Chicago Medical Journal*, and notice the following editorial remarks upon the proposed meeting, indicating that we have no ground for expecting either co-operation of the members from the South, or harmony of action at home. If the profession of Chicago are not prepared to receive the Association, this will constitute the best possible ground for delay or postponement; of this we shall be hereafter informed. In order to show the case more fully and allow our readers to judge for themselves, we copy the remarks of the editor of the *Chicago Medical Journal*, a distinguished member of the profession in that city:

"PROPOSED MEETING OF THE AMERICAN MEDICAL ASSOCIATION.—We received too late for insertion in the last number, and after the notice which it contained had been written, a call signed by Dr. N. S. Davis, and purporting to be authorized by the "Committee of Arrangements," for a meeting of the American Medical Association, to take place in Chicago on the first Tuesday in June, 1863. On enquiry of some members of said Committee we find they know nothing about said call. The Committee without any authority adjourned the meeting of 1861–1862; and now some of them, equally without authority, have announced a meeting under circumstances quite as unfavorable as have existed in former years.

If a meeting is desired we conceive it devolved upon the State Societies, and other organized bodies, including teachers in Colleges and Hospitals, to intimate their wishes to that effect. This, as far as we are informed, has not been done in more than one instance.

The Association meeting in June can have no claim to be considered "National," and must if convened but favor the effect of forever perpetuating its sectional character. For ourselves we do not despair of the return of better days, when our brethren now in the army and numerous Union men in the Southern States shall join with their brethren in the North in meetings truly National in their character. We know that some most active in the matter do not share this hope, probably do not desire such a result, but it is a conviction to which we shall cling to the last.

The excitement of civil war is not favorable to the calmness required in scientific meetings. The National Association of Science has thought it wise to suspend its annual assemblage. Several of the State Societies have followed its example. From such no representatives can be expected.

The time appointed is peculiarly unfortuate, being the day on which a great "Canal Convention" is to convene in this city. Citizens will be called upon to open their houses for the accommodation of the members of this latter body. Several eminent medical gentlemen will find it necessary to devote most of their time to it. Many will desire to be present to listen to its proceedings. If, therefore, a general attendance is desired, we would suggest that the second Tuesday of June be substituted for the first Tuesday. We make this suggestion in the interest of the profession of the city who will desire to give to their friends a cordial and hospitable reception whenever they may do us the honor of a visit. D. B."

